# A Korean version of the Oral Impacts on Daily Performances (OIDP) scale in elderly populations: Validity, reliability and prevalence

**DOI:** 10.1186/1477-7525-6-17

**Published:** 2008-02-27

**Authors:** Se-Hwan Jung, Jae-In Ryu, Georgios Tsakos, Aubrey Sheiham

**Affiliations:** 1 Department of Preventive and Public Health Dentistry, Kangnung National University, Gangneung, Republic of Korea; 2 Department of Epidemiology and Public Health, University College London, London, UK

## Abstract

**Background:**

This study aimed to develop a Korean version of the OIDP index for elderly people and to assess the levels of sociodental impacts in an older Korean population.

**Methods:**

The OIDP index for elderly people was cross-culturally adapted from English into Korean and then the derived instrument was tested for reliability and validity. The study population was elderly (65+ year-old) residents of Gangneung City, South Korea. Twenty two of the 222 senior day centres were randomly selected.

**Results:**

687 people were invited and 668 participated in the study (response rate: 97.2%). The standardized Cronbach's alpha coefficient was 0.85. The OIDP related significantly with different subjective measures of oral and general health (p < 0.001). 62.9% of the people had oral impacts relating to one or more performances, with eating food being the most frequently affected performance (47.6%). More than 70% of people with oral impacts had up to 3 performances affected by oral health conditions.

**Conclusion:**

The Korean OIDP index showed satisfactory validity and internal consistency reliability, confirming its appropriateness for use among older Korean people. The prevalence of oral health related impacts was high. Future studies should focus on the test-retest reliability and the sensitivity to change of the Korean OIDP.

## Background

National dental surveys aim to provide planners and policy makers with sound data for planning dental services. Unfortunately, most surveys only use conventional normative oral health indices [1,2]. Such clinical indicators frequently overestimate oral health needs [3]. More comprehensive measures, including socio-dental indicators which have been developed to overcome this problem [4], should be used. They complement clinical measurements and measure a range of dimensions of Oral Health-Related Quality of Life (OHRQoL) including oral health impairments, functional limitation, and disability [2,5,6]. The virtue of OHRQoL measures is that they highlight the importance of the oral impacts and give planners insights into the subjective feelings of the population.

The Oral Impacts on Daily Performance (OIDP) [7] is one of the most widely used sociodental indicators. The theoretical framework on which the OIDP is based is modified from the WHO's [8] International Classification of Impairments, Disabilities and Handicaps [9]. The main modification is that different levels of the concepts are established: oral status or impairments, intermediate impacts (pain, discomfort, functional limitation or dissatisfaction with appearance), and ultimate impacts which cover the concepts of disability and handicap. The OIDP focuses on the third level of measurement, thus assessing oral impacts on the ability to perform daily activities. It is easy to use and has been successfully tested for reliability and validity in adult and elderly populations in different settings [7,10-15]. Before using an OHRQoL measure in a new setting, it is necessary to re-establish its psychometric properties. Therefore, the first objective of this study is to develop and validate a Korean version of the OIDP index for elderly people. The second objective is to assess the levels of sociodental impacts in an older Korean population.

## Methods

The research sites for this study were located in Gangneung City, the North-East of South Korea. The study population was residents of this city, aged over 65 years. The sampling frame referred to the 222 senior day centres of the city. These are places where retired free-living senior adults meet, communicate and participate in recreational activities. Twenty two of the 222 senior day centres were randomly selected for this study.

To cross-culturally adapt the OIDP for use among Korean elderly people, the English OIDP index for elderly people [15] was first translated into Korean by three independently working Korean scholars with a good knowledge of English. Because 19.2% of the elderly are illiterate in Korea [16], the Korean version of the OIDP index needed to be a questionnaire for face to face interviews. The translated questionnaire was first tested on 20 people in a senior day centre. After the interviews, the interviewers had informal conversations with the participants about the questionnaire. Minor modifications were made to the format of questionnaires and to the wording. Secondly, the backward translation of the draft version of the Korean OIDP into English was carried out by experts in foreign languages at the Institute of Kangnung National University in Korea. Then, experts on dental public health, language and translation compared the back translation with the original English version of questionnaire. Finally, the backward translation of English OIDP was verified with the original developers of the OIDP at University College London.

A second pilot study was carried out prior to the main study to test the feasibility of questionnaire administration under field conditions, as well as the understanding of the content of the questionnaire under investigation. Another 20 elderly people from a senior day centre participated in this pilot testing of the oral health assessment and questionnaire forms. The pilot study confirmed the feasibility of the methodology with some modifications.

An examiner was trained and calibrated to the 2000 and 2003 National Oral Health Survey. He was fully aware of the form and criteria for the oral examination of National Survey. The interviewers were briefed about the purpose and process of the study. They had experience in interviewing people for oral health related questionnaires and had worked in Kangnung Dental College as researchers. The manual for the interviewers of the OIDP was completed based on the discussion from pilot tests.

The main study was implemented on elderly people attending 22 senior day centres. Information letters were sent to the selected senior day centres at least one week prior to the date of the data collection. Data were collected using the clinical oral health assessment form and the oral health questionnaires. Clinical dental examinations were used to determine whether subjects were dentate or edentulous, count the number of natural teeth present and assess the need for restorative and prosthetic treatment. The oral health questionnaire recorded data on demographic information, perceived oral health conditions, satisfaction with oral health status, perceived general health conditions, and pain using a visual analogue scale (VAS). A Visual Analogue Scale is a measurement instrument that tries to measure a characteristic or attitude that is believed to range across a continuum of values and cannot easily be directly measured [17].

The study was ethically approved by the Institutional Review Board (IRB) in Kangnung National University Dental College (IRB Letter No. 2004-02). Individual positive consent was sought for the clinical oral examination and the questionnaire-led interview. Subjects were free to withdraw from the study at any stage. Local city councils and health authorities in the study areas were contacted to gain permission and co-operation. Every participant received information on their measured oral and general health conditions.

### Data analysis

Each performance score of OIDP was calculated by multiplying frequency (0–5) and severity scores (0–5). Then these scores for the 10 performances were summed up. Finally the overall OIDP score was the sum divided by maximum possible score (5 frequency × 5 severity scores × 10 performances) and multiplied with 100 to give a percentage score. In addition to the OIDP score, the extent of OIDP impacts was also reported. This is an alternative way of quantifying oral impacts, previously suggested for the OIDP [18] and used in the Child-OIDP [19], and refers to the number of OIDP performances with impacts (PWI) affecting a respondent's quality of life over the past six months.

Face and content validity were tested in the pilot study with regard to content, wording, scoring method, and easiness and appropriateness of the questionnaire administration. Content validity measures whether the components of the scale or item cover all aspects of the attribute to be measured or the content of the variables matches the name which it has been given [20]. The criterion validity is defined as the correlation of a scale with some other measure of the trait under study, ideally a 'gold standard', while the construct validity, described as probably the most important approach to validity [21,22], tests logical constructs by assessing the relationship of the instrument under test with measures of other related constructs. As the OIDP is intended to be used in dental needs assessment, perceived need for dental treatment was chosen as a proxy measure for the criterion validity testing, while pain visual analogue scales, perceived oral health status, satisfaction with oral health status and perceived general health status for the construct validity testing [23] of the Korean OIDP. Due to their skewed frequency distribution, the pain VAS scores were categorised into three, none (0), low (1–5), and high (6–10) pain experience. The OIDP scores were not normally distributed and the Kruskal-Wallis test was used for analyzing the relationship between OIDP scores and subjective questions. In addition, the Spearman's rank correlation coefficient was used for the association between the OIDP scores and the pain VAS scores. Internal reliability of the OIDP was tested by inter-item correlations, corrected item-total correlations, standardised alpha coefficient and alpha if item deleted [24]. Finally, the relationships between the OIDP scores and clinical measures of restorative and prosthetic treatment needs were assessed through the use of Kruskal-Wallis test. Treatment need variables were categorised into three groups: 1) restorative treatment needs: no need, one surface restoration, pulp care with restoration or extraction; and 2) prosthetic treatment needs: no need, need only in one jaw, needs in both jaws. SPSS version 13.0 for Windows was used for the analysis of data in this sample. The cut-off level for statistical significance was 0.05 [25].

## Results

687 people were invited to participate in this study and 668 agreed with a response rate of 97.2%. The sociodemographic distribution of the sample is shown in Table 1. The age ranged from 65 to 93 and the mean of it was 75.5 ± 6.0 years. There were slightly more females than males. Most participants had not finished elementary school (86%). More than half of the people had dentulous dentitions in both jaws and around a quarter of the study sample was totally edentulous (Table 2). The mean number of permanent natural teeth present was 11.6 ± 9.8. Clinically 36.4% of subjects were assessed to need restorative treatment, while in terms of prosthetic treatment 23.5% of needed treatment in one jaw and 20.7% in both jaws. 64.6% of the respondents thought they need dental treatments and 62.4% answered that they have poor oral condition. Slightly less than half of the subjects (43.1%) were satisfied with their oral conditions.

**Table 1:** Socio-demographic characteristics of the Korean elderly subjects (n = 668)

**Demographic information**		**Percent**
Age	65–74 years	46.7
	over 75 years	53.3
Sex	male	49.1
	female	50.9
Household status	living alone	23.1
	living with your husband/wife	42.8
	living with children	33.7
	other	0.4
Education	no formal education	43.0
	primary school	42.8
	middle school	6.7
	high school	6.4
	college	1.0

**Table 2:** Percentage distribution of clinical and subjective status in Korean elderly subjects (n = 668)

**Clinical status and needs**	**Categories**	**Percent**
Denture status	both edentulous	22.5
	upper edentulous	14.7
	lower edentulous	3.7
	both dentulous	59.1

Number of natural teeth	0	22.5
	1–10	27.2
	11–20	23.8
	21 or more	26.5

Restorative need	no need	63.6
	one surface	7.7
	pulp care + restoration or extraction	28.7

Prosthetic need	no need	55.8
	in one jaw	23.5
	both jaws	20.7

**Subjective status and needs**	**Categories**	**Percent**

Perceived dental treatment need^1^	no need at all	8.9
	no need	26.4
	fairly need	9.8
	need	29.0
	high level of need	25.8

Perceived oral health ^2^	good	18.5
	fair	19.1
	poor	34.6
	very poor	27.8

Pain VAS (categorised)	0	65.4
	1–5	21.0
	6–10	13.6

Satisfaction with oral health status ^3^	satisfied	23.4
	fairly satisfied	19.7
	not satisfied	31.1
	not at all satisfied	25.8

Perceived general health ^3^	good	18.0
	fair	25,2
	poor	30.0
	very poor	26.7

^1 ^Due to missing cases, analysis carried out on 651 people.

^2 ^Due to missing cases, analysis carried out on 665 people.

^3 ^Due to missing cases, analysis carried out on 666 people.

The criterion and construct validity of OIDP index were assessed through its association with several subjective health status variables (Table 3). Participants with perceived needs for dental treatment had much higher OIDP scores than those who did not have perceived need for treatment (p < 0.001). Similarly, people who reported worse oral health perceptions or had higher pain VAS scores had significantly higher OIDP scores than their counterparts with better oral health perceptions or lower pain scores respectively (p < 0.001 for both). The association between pain VAS score and the OIDP score was also significant (p < 0.001), with a relatively strong correlation coefficient of 0.42. In relation to construct validity tests, people with higher levels of satisfaction with oral health and perceived general health status had lower OIDP scores than those with lower levels of satisfaction and perceived general health status respectively (p < 0.001). All those relationships showed a clear trend with OIDP scores, not only a difference between the extreme groups; the worse the perception, the higher the OIDP score, which indicates higher level of oral impacts.

**Table 3:** Criterion and construct validity tests for Korean elderly OIDP index and relationship with clinical measures: OIDP scores (0–100) between different categories of outcome measurements

**Variables**	**Categories**	**N**	**Mean**	**(SD)**	**Quartiles**	**P**
**Subjective health status measures**						

Perceived dental treatment need (n = 651)	no need at all	58	0.9	(2.8)	(0.0, 0.0, 0.0)	< 0.001
	no need	172	3.4	(9.1)	(0.0, 0.0, 2.2)	
	fair level of need	64	3.6	(7.3)	(0.0, 0.0, 3.2)	
	need	189	6.8	(11.0)	(0.0, 1.8, 8.6)	
	high level of need	168	17.8	(20.1)	(2.7, 10.2, 25.4)	

Perceived oral health (n = 665)	good	123	1.9	(6.2)	(0.0, 0.0, 0.0)	< 0.001
	fair	127	2.3	(4.9)	(0.0, 0.0, 1.6)	
	poor	230	7.7	(13.1)	(0.0, 2.2, 9.6)	
	very poor	185	15.8	(19.1)	(0.4, 10.0, 20.6)	

Pain VAS (categorised) (n = 668)	0	437	4.5	(10.2)	(0.0, 0.0, 6.0)	< 0.001
	1–5	140	8.7	(13.2)	(0.0, 2.8, 12.0)	
	6–10	91	22.1	(21.3)	(6.0, 17.6, 31.2)	

Satisfaction with oral health (n = 666)	satisfied	156	1.8	(6.7)	(0.0, 0.0, 0.0)	< 0.001
	fairly satisfied	131	2.5	(5.7)	(0.0, 0.0, 1.6)	
	not satisfied	207	9.0	(13.6)	(0.0, 4.0, 12.0)	
	not at all satisfied	172	16.1	(19.2)	(1.7, 10.0, 20.4)	

Perceived general health (n = 666)	good	120	3.4	(8.2)	(0.0, 0.0, 2.0)	< 0.001
	fair	168	7.4	(14.5)	(0.0, 0.0, 8.3)	
	poor	200	7.7	(14.6)	(0.0, 0.6, 8.4)	
	very poor	178	11.3	(15.7)	(0.0, 6.0, 16.0)	

**Clinical dental treatment needs**						

Restorative need	no need	425	7.1	(13.4)	(0.0, 0.0, 6.0)	= 0.016
	one surface	51	7.2	(13.2)	(0.0, 2.0, 13.4)	
	pulp care + restoration or extraction	192	9.6	(15.8)	(0.0, 3.0, 14.0)	

Prosthetic need	no need	373	6.3	(13.1)	(0.0, 0.0, 8.0)	< 0.001
	in one jaw	157	9.0	(14.4)	(0.0, 0.0, 10.0)	
	both jaws	138	10.4	(16.0)	(0.0, 2.4, 12.0)	

In addition, the OIDP was able to discriminate between participants with different degrees of treatment needs. The relationship between the OIDP score and restorative treatment need showed significant trend (p = 0.016), with worse OHRQoL among participants in higher need for treatment. Furthermore, the relationship between OIDP score and prosthetic treatment need of participants showed a similar pattern (p < 0.001); the mean OIDP score among subjects with no prosthetic need was 6.3, while the figures among those with need in one jaw and need in both jaws were 9.0 and 10.4 respectively.

Table 4 shows that the inter-item correlation coefficients among the 10 items scores of the OIDP index ranged from 0.13 to 0.74. None of the scores were negative suggesting that the items were homogenous. Also, the correlations were not high enough for any item to be redundant. Corrected item-total correlations coefficients ranged from 0.40 to 0.68 (Table 5). Cronbach's alpha coefficient was 0.84 and the standardized alpha was 0.85. When any of the items was deleted the alpha coefficient did not override the standardized alpha.

**Table 4:** Reliability analysis of OIDP index for Korean participants: OIDP items Correlation matrix

	Performances									
Performances	1.	2.	3.	4.	5.	6.	7.	8.	9.	10.
1. eating	1.00									
2. speaking	0.39	1.00								
3. smiling	0.30	0.48	1.00							
4. light physical activities	0.39	0.35	0.22	1.00						
5. daily activities	0.38	0.40	0.38	0.73	1.00					
6. enjoying contact	0.37	0.53	0.48	0.52	0.74	1.00				
7. cleaning teeth	0.37	0.26	0.30	0.13	0.21	0.21	1.00			
8. sleeping	0.27	0.25	0.16	0.26	0.32	0.29	0.29	1.00		
9. relaxing	0.34	0.21	0.21	0.42	0.37	0.35	0.28	0.41	1.00	
10. emotional stability	0.53	0.44	0.43	0.49	0.48	0.54	0.32	0.30	0.42	1.00

**Table 5:** Reliability analysis of OIDP index: Corrected item-total correlation, Cronbach's Alpha, Standardised Alpha and Alpha if item deleted

**Performances**	**Corrected item-total correlation**	**Alpha if item deleted**
1. eating	0.57	0.83
2. speaking	0.57	0.83
3. smiling	0.51	0.83
4. light physical activities	0.58	0.83
5. daily activities	0.67	0.82
6. enjoying contact	0.68	0.82
7. cleaning teeth	0.40	0.84
8. sleeping	0.41	0.84
9. relaxing	0.48	0.84
10. emotional stability	0.68	0.81
		
Alpha	= 0.84	
Standardised item Alpha	= 0.85	

**Figure 1:**
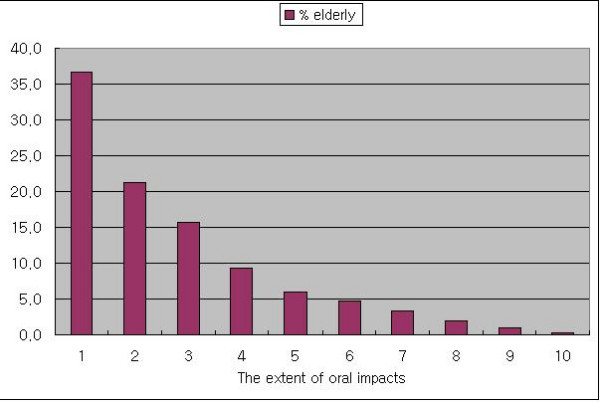
Percentage distribution of the number of OIDP performances affected among those with any impact.

A relatively high percentage of people (62.9%) reported oral impacts relating to one or more performances. The distribution of people with oral impacts for the different performances is shown in Table 6. The most frequently affected performance was eating food (47.6%). The next most common impacts related to speaking (24.9%) and cleaning teeth (19.3%). The extent of oral impacts ranged from 0 to 10 performances with impacts (PWIs). More than 70% of people with oral impacts had up to three performances affected from oral conditions; 36.7% had 1 PWI, 21.2% had 2 PWIs, and 15.7% had 3 PWIs (Figure 1).

**Table 6:** Percentage distribution of people with positive OIDP impacts (n = 668)

Positive OIDP performance	**N**	**%**
Any performance affected	420	62.9
Eating	318	47.6
Speaking	166	24.9
Smiling	97	14.5
Light physical activities (housework)	56	8.4
Daily activities (going out)	64	9.6
Enjoying contact	72	10.8
Cleaning teeth	129	19.3
Sleeping	65	9.7
Relaxing	44	6.6
Emotional stability	128	19.2

## Discussion

This is the first study to adapt the OIDP index in Korean and test its validity and reliability on an elderly Korean population. Considerable efforts were devoted to the appropriate cross-cultural adaptation of the instrument, in order to overcome the language and cultural differences. Failure to deal with those issues can raise critical questions on the validity of an adapted version of an index [26]. Structured translation is one of the most important procedures to avoid this problem. This study followed the methodology from previous studies on the OIDP [6,12,15,27,28]. The professional language unit, consisting of staff who can speak both English and Korean fluently, undertook the forward and backward translation. After that the draft was re-examined twice in pilot studies.

The pilot study provided insights into the understanding of Korean version of OIDP by Koreans. For example, they confused the meaning of 'sleeping' because it could be interpreted as 'sleeping' or 'going to bed' which are the same word in Korean. 'Sleeping' was complemented with extra explanation of this performance. Second, some of them had difficulties exactly setting the time frame for the reporting of oral impacts when the wording 'in the past 6 months' was used. As a solution the interviewers mentioned the exact month that corresponded to the six months prior to the main study. Those minor modifications improved understanding of the questionnaires and all participants in the main study answered without missing out any item.

The validity of quality of life mainly relied on the subjective measurement. The rationale for this comes from the conceptual distinction between health and disease [15,29]. A normative index measures only biological pathology, without considering social and psychological aspects of health. There are well known limitations of the normative assessment of oral health and needs [30]. Disease does not always negatively affect subjective perceptions of wellbeing [15]. Consequently, clinical oral health indicators "tell us nothing about the functioning of either the oral cavity or the person as a whole and nothing about subjectively perceived symptoms such as pain and discomfort" [31]. In addition to using the relationship between OIDP and subjective health status measures for the core assessment of validity, the ability of the index was also assessed to discriminate between different clinical status groups.

The results of this study showed that the Korean OIDP for elderly people is a valid and reliable instrument to measure OHRQoL. Face and content validity were confirmed in the pilot study. In this study, all examined relationships between OIDP score and subjective oral health measures (perceived dental treatment need, perceived oral health condition, pain visual analogue scale, satisfaction with oral health) were statistically significant and showed a clear trend in the expected direction; the worse the subjective oral health rating, the higher the OIDP score. Furthermore, in line with previous studies [15,32,33], the OIDP score was significantly associated with perceived general health. In addition to using the relationship between OIDP and subjective health status measures for the core assessment of validity, the ability of the index to discriminate between different clinical groups was successfully assessed. Indeed, the OIDP was able to discriminate between groups with different degrees of treatment need. Subjects with no need for dental treatment reported significantly lower levels of oral impacts than their counterparts with low levels of treatment needs, and those, in turn, had lower levels of impacts than subjects with higher levels of treatment needs. This significant trend was observed in relation to both restorative and prosthetic treatment needs.

Inter-item correlation, corrected item-total correlation, and Cronbach's alpha indicated this index had excellent internal consistency. None of the inter-item correlations was negative and all item-total correlations were above the minimum recommended level of 0.20 [34] for including an item in a scale, hence demonstrating the homogeneity of the items. Furthermore, Cronbach's alpha was much higher than the recommended thresholds for research purposes and studying groups and higher than previous studies in other settings [7,15,27].

The overall prevalence of Oral Impacts on Daily Performance (OIDP) was 63%. That was similar to Tanzanian (62.1%) [13] and Thai (52.8%) [14] studies on a similar age group but higher than in other countries [7,15,27,35]. The differences in prevalence may be related to cultural differences. The comparison of oral health care systems in six countries reported similar results [36]. There were big differences in subjective oral health conditions. For example, people in Japan, which is close to Korea, were more likely to answer that their oral conditions were not good. It is possible that the cultural gap between western and eastern countries is responsible for the differences. Eating was the most prevalent performance affected by oral impacts among the ten items. That was consistent with the results in other studies [6,11,13-15,19,27,35,37]. The performance with the lowest prevalence of oral impacts was relaxing (7%). Other studies suggested excluding two items such as cleaning and light physical activities which showed the extremely low prevalence from the OIDP [15]. However, our results justify the inclusion of all ten items in the Korean OIDP in future studies, as no item had extremely low prevalence.

The study used simple random sampling from the senior day centres in Gangneung city. The sample may not represent the whole population of Korea. However, the demographic characteristics of this study sample were broadly similar to the rural elderly population in the National Survey, with slightly lower education and income level [16]. The response rate was very high (97.2%). This was partly facilitated by the considerable administrative support from the Gangneung City council and health centres, especially as this study was part of a broader endeavour to develop a public oral health plan for the elderly in the area. In addition, study subjects received a free medical and dental examination, which also acted as an incentive to participate. Future studies should also evaluate the test-retest reliability of the Korean OIDP, while longitudinal studies would allow for testing its sensitivity to change. Finally, the national Korean oral health survey should include a subjective measure of oral impacts as one of its health measures.

## Conclusion

In conclusion, the Korean OIDP index showed valid and reliable psychometric properties, confirming its appropriateness to measure the OHRQoL of older Korean people. The prevalence of oral impacts was high, with eating food being the most frequently affected performance. Future studies should focus on the test-retest reliability and the sensitivity to change of the Korean OIDP.

## Abbreviations

Oral Impacts on Daily Performances (OIDP); Oral Health-Related Quality of Life (OHRQoL); Visual Analogue Scale (VAS); Institutional Review Board (IRB); Performances with Impacts (PWIs)

## Competing interests

The author(s) declare that they have no competing interests.

## Authors' contributions

SHJ contributed to make a conception and design of the study, acquisition of data, analysis and interpretation of data, and drafting the manuscript.

JIR participated in a conception and designing of the study, analysis and interpretation of the data, and drafting the manuscript.

GT advised on the study design and analysis and participated in the interpretation of the data and discussion of the findings.

AS advised on the study design and analysis and participated in the interpretation of the data and discussion of the findings.

All authors read and approved the final manuscript.
